# 5-(4-Fluoro­phen­yl)-3-[5-methyl-1-(4-methyl­phen­yl)-1*H*-1,2,3-triazol-4-yl]-4,5-dihydro-1*H*-pyrazole-1-carbothio­amide

**DOI:** 10.1107/S1600536812024245

**Published:** 2012-05-31

**Authors:** Bakr F. Abdel-Wahab, Ehab Abdel-Latif, Seik Weng Ng, Edward R. T. Tiekink

**Affiliations:** a Applied Organic Chemistry Department, National Research Centre, Dokki, 12622 Giza, Egypt; b Department of Chemistry, Faculty of Science, Mansoura University, ET-35516 Mansoura, Egypt; c Department of Chemistry, University of Malaya, 50603 Kuala Lumpur, Malaysia; d Chemistry Department, Faculty of Science, King Abdulaziz University, PO Box 80203 Jeddah, Saudi Arabia

## Abstract

In the title compound, C_20_H_19_FN_6_S, the pyrazole ring has an envelope conformation, with the methine C atom being the flap atom. The dihedral angle between the least-squares plane through the pyrazole and triazole rings is 7.59 (9)°, and the triazole and attached benzene ring form a dihedral angle of 74.79 (9)°. The thio­urea group is coplanar with the pyrazole ring [N—N—C—S torsion angle = −179.93 (11)°], which enables the formation of an intra­molecular N—H⋯N hydrogen bond. In the crystal, inversion-related mol­ecules associate *via* N—H⋯S hydrogen bonds and eight-membered {⋯HNCS}_2_ synthons feature in the crystal packing. These synthons are connected into supra­molecular chains along the *a* axis *via* N—H⋯F hydrogen bonds, and the chains are consolidated into layers in the *ab* plane *via* C—H⋯S and C—H⋯F contacts.

## Related literature

For the biological activity of pyrazolyl-1,2,3-triazoles, see: Abdel-Wahab *et al.* (2012*a* ▶); Booth & Ross (1982 ▶); Curran (1982 ▶). For a related pyrazolyl-1,2,3-triazole structure, see: Abdel-Wahab *et al.* (2012*b* ▶).
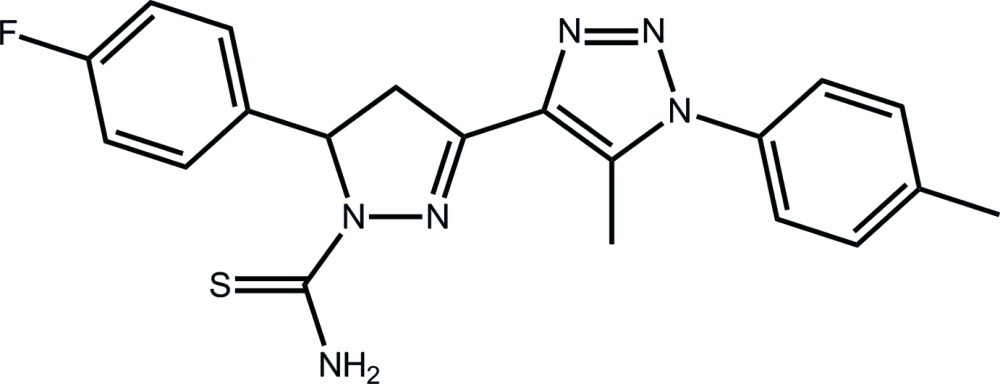


## Experimental

### 

#### Crystal data

C_20_H_19_FN_6_S
*M* *_r_* = 394.47
Monoclinic, 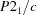

*a* = 9.4388 (4) Å
*b* = 6.5476 (3) Å
*c* = 32.1483 (18) Å
β = 91.288 (4)°
*V* = 1986.31 (17) Å^3^
*Z* = 4
Mo *K*α radiation
μ = 0.19 mm^−1^
*T* = 100 K
0.40 × 0.30 × 0.20 mm

#### Data collection

Agilent SuperNova Dual diffractometer with an Atlas detector
Absorption correction: multi-scan (*CrysAlis PRO*; Agilent, 2011 ▶) *T*_min_ = 0.855, *T*_max_ = 1.000
7765 measured reflections
4551 independent reflections
3809 reflections with *I* > 2σ(*I*)
*R*_int_ = 0.027

#### Refinement

*R*[*F* ^2^ > 2σ(*F* ^2^)] = 0.043
*wR*(*F* ^2^) = 0.109
*S* = 1.02
4551 reflections
263 parameters
H atoms treated by a mixture of independent and constrained refinement
Δρ_max_ = 0.64 e Å^−3^
Δρ_min_ = −0.36 e Å^−3^

### 

Data collection: *CrysAlis PRO* (Agilent, 2011 ▶); cell refinement: *CrysAlis PRO*; data reduction: *CrysAlis PRO*; program(s) used to solve structure: *SHELXS97* (Sheldrick, 2008 ▶); program(s) used to refine structure: *SHELXL97* (Sheldrick, 2008 ▶); molecular graphics: *ORTEP-3* (Farrugia, 1997 ▶) and *DIAMOND* (Brandenburg, 2006 ▶); software used to prepare material for publication: *publCIF* (Westrip, 2010 ▶).

## Supplementary Material

Crystal structure: contains datablock(s) global, I. DOI: 10.1107/S1600536812024245/su2439sup1.cif

Structure factors: contains datablock(s) I. DOI: 10.1107/S1600536812024245/su2439Isup2.hkl

Supplementary material file. DOI: 10.1107/S1600536812024245/su2439Isup3.cml

Additional supplementary materials:  crystallographic information; 3D view; checkCIF report

## Figures and Tables

**Table 1:** Hydrogen-bond geometry (Å, °)

*D*—H⋯*A*	*D*—H	H⋯*A*	*D*⋯*A*	*D*—H⋯*A*
N1—H1N⋯S1^i^	0.89 (2)	2.432 (19)	3.3159 (14)	172.7 (16)
N1—H2N⋯F1^ii^	0.86 (2)	2.29 (2)	2.9940 (18)	138.9 (17)
N1—H2N⋯N3	0.86 (2)	2.30 (2)	2.6554 (19)	104.9 (15)
C3—H3*A*⋯S1^iii^	0.99	2.87	3.8390 (19)	166
C9—H9⋯S1^iv^	0.95	2.83	3.5595 (18)	135
C15—H15⋯F1^v^	0.95	2.41	3.2502 (19)	148

Symmetry codes: (i) 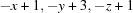; (ii) 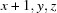; (iii) 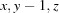; (iv) 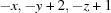; (v) 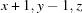.

**Fig. 1.**
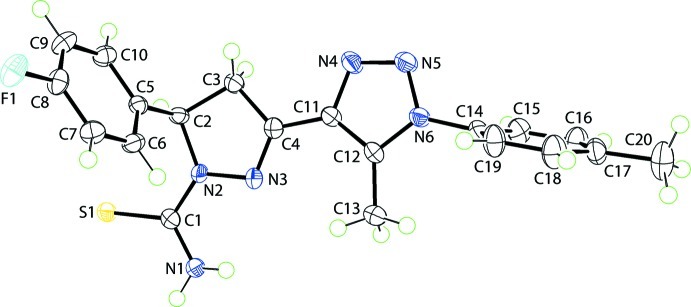
The molecular structure of (I) showing the atom-labelling scheme and displacement ellipsoids at the 70% probability level.

**Fig. 2.**
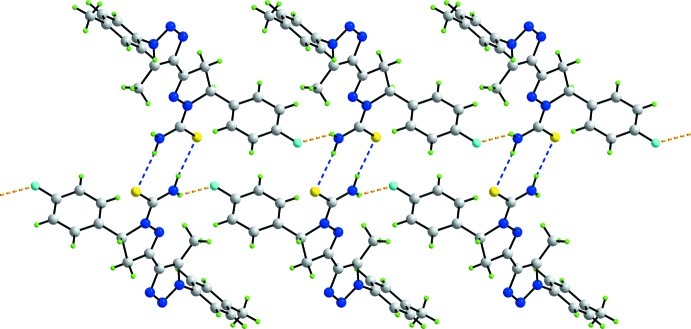
A view of a supramolecular chain along the a axis in (I). The N—H···S and N—H···F hydrogen bonds are shown as blue and orange dashed lines, respectively.

**Fig. 3.**
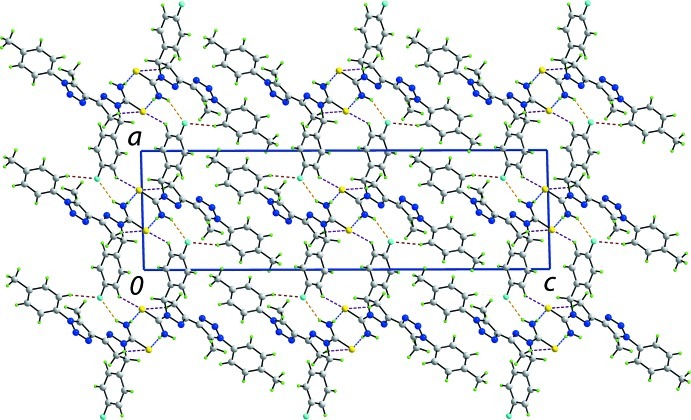
A view in projection down the b axis of the unit-cell contents for (I) highlighting the inter-digitation of layers along the c axis. The N—H···S, N—H···F, C—H···S and C—H···F interactions are shown as blue, orange, purple and brown dashed lines, respectively.

## supplementary crystallographic information

### Comment

In continuation of structural studies of related drug candidates (Abdel-Wahab *et al.*, 2012*b*), the title compound, (I), was investigated crystallographically. This compound is of interest owing to the established biological activities exhibited by pyrazolyl-1,2,3-triazoles (Abdel-Wahab *et al.*, 2012*a*; Booth & Ross, 1982; Curran, 1982).

The pyrazole ring in (I), Fig. 1, adopts an envelope conformation (r.m.s. deviation = 0.138 Å) with the methine-C2 atom being the flap atom. The dihedral angle between the least-squares plane through this ring and the adjacent triazole ring is 7.59 (9)°. The benzene ring connected to the triazole ring is twisted out of its plane, forming a dihedral angle of 74.79 (9)°. The N3—N2—C1—S1 torsion angle of -179.93 (11)° indicates that the thiourea moiety is coplanar with the pyrazole ring. This arrangement coupled with the orientation of the amino group towards the ring enables the formation of an intramolecular N—H···N hydrogen bond (Table 1).

In the crystal, centrosymmetrically related molecules associate *via* N—H···S hydrogen bonds and eight-membered {···HNCS}_2_ synthons feature in the crystal packing (Table 1). These are connected into supramolecular chains along the *a* axis *via* N—H···F hydrogen bonds (Fig. 2 and Table 1). Chains are connected into layers in the *ab* plane *via* C—H···S and C—H···F contacts (Table 1). Layers inter-digitate along the *c* axis with no specific interactions between them (Fig. 3).

### Experimental

The title compound was prepared according to the reported method (Abdel-Wahab *et al.*, 2012*a*). Crystals were obtained from its DMF solution by slow evaporation at room temperature.

### Refinement

C-bound H atoms were placed in calculated positions [C—H = 0.95 to 1.00 Å, *U*_iso_(H) = 1.2*U*_eq_(C) or 1.5*U*_eq_(C) for methyl H atoms] and were included in the refinement in the riding model approximation. The N-bound H atoms were freely refined.

### Crystal data

**Table:**

C_20_H_19_FN_6_S	*F*(000) = 824
*M**_r_* = 394.47	*D*_x_ = 1.319 Mg m^−^^3^
Monoclinic, *P*2_1_/*c*	Mo *K*α radiation, λ = 0.71073 Å
Hall symbol: -P 2ybc	Cell parameters from 3598 reflections
*a* = 9.4388 (4) Å	θ = 2.2–27.5°
*b* = 6.5476 (3) Å	µ = 0.19 mm^−^^1^
*c* = 32.1483 (18) Å	*T* = 100 K
β = 91.288 (4)°	Prism, light-brown
*V* = 1986.31 (17) Å^3^	0.40 × 0.30 × 0.20 mm
*Z* = 4	

### Data collection

**Table:**

Agilent SuperNova Dual diffractometer with an Atlas detector	4551 independent reflections
Radiation source: SuperNova (Mo) X-ray Source	3809 reflections with *I* > 2σ(*I*)
Mirror monochromator	*R*_int_ = 0.027
Detector resolution: 10.4041 pixels mm^-1^	θ_max_ = 27.6°, θ_min_ = 2.5°
ω scans	*h* = −11→12
Absorption correction: multi-scan (*CrysAlis PRO*; Agilent, 2011)	*k* = −8→8
*T*_min_ = 0.855, *T*_max_ = 1.000	*l* = −23→41
7765 measured reflections	

### Refinement

**Table:**

Refinement on *F*^2^	Primary atom site location: structure-invariant direct methods
Least-squares matrix: full	Secondary atom site location: difference Fourier map
*R*[*F*^2^ > 2σ(*F*^2^)] = 0.043	Hydrogen site location: inferred from neighbouring sites
*wR*(*F*^2^) = 0.109	H atoms treated by a mixture of independent and constrained refinement
*S* = 1.02	*w* = 1/[σ^2^(*F*_o_^2^) + (0.045*P*)^2^ + 1.138*P*] where *P* = (*F*_o_^2^ + 2*F*_c_^2^)/3
4551 reflections	(Δ/σ)_max_ = 0.002
263 parameters	Δρ_max_ = 0.64 e Å^−^^3^
0 restraints	Δρ_min_ = −0.36 e Å^−^^3^

### Special details

**Table:**

Geometry. All s.u.'s (except the s.u. in the dihedral angle between two l.s. planes) are estimated using the full covariance matrix. The cell s.u.'s are taken into account individually in the estimation of s.u.'s in distances, angles and torsion angles; correlations between s.u.'s in cell parameters are only used when they are defined by crystal symmetry. An approximate (isotropic) treatment of cell s.u.'s is used for estimating s.u.'s involving l.s. planes.
Refinement. Refinement of *F*^2^ against ALL reflections. The weighted *R*-factor *wR* and goodness of fit *S* are based on *F*^2^, conventional *R*-factors *R* are based on *F*, with *F* set to zero for negative *F*^2^. The threshold expression of *F*^2^ > σ(*F*^2^) is used only for calculating *R*-factors(gt) *etc*. and is not relevant to the choice of reflections for refinement. *R*-factors based on *F*^2^ are statistically about twice as large as those based on *F*, and *R*-factors based on ALL data will be even larger.

### Fractional atomic coordinates and isotropic or equivalent isotropic displacement parameters (Å^2^)

**Table:**

	*x*	*y*	*z*	*U*_iso_*/*U*_eq_	
S1	0.32997 (4)	1.28074 (6)	0.507140 (13)	0.01661 (12)	
F1	−0.23235 (10)	1.32770 (17)	0.39047 (3)	0.0253 (3)	
N1	0.53882 (15)	1.2902 (2)	0.45302 (5)	0.0181 (3)	
N2	0.38250 (13)	1.0266 (2)	0.44567 (4)	0.0137 (3)	
N3	0.45951 (13)	0.9562 (2)	0.41184 (4)	0.0145 (3)	
N4	0.40790 (15)	0.4715 (2)	0.35943 (5)	0.0212 (3)	
N5	0.47668 (16)	0.3904 (2)	0.32855 (5)	0.0224 (3)	
N6	0.57249 (14)	0.5319 (2)	0.31626 (4)	0.0161 (3)	
C1	0.42324 (16)	1.1970 (2)	0.46636 (5)	0.0139 (3)	
C2	0.25153 (16)	0.9101 (2)	0.45166 (5)	0.0139 (3)	
H2	0.2393	0.8789	0.4818	0.017*	
C3	0.28474 (17)	0.7137 (3)	0.42742 (5)	0.0162 (3)	
H3A	0.3146	0.6017	0.4463	0.019*	
H3B	0.2023	0.6684	0.4102	0.019*	
C4	0.40479 (16)	0.7836 (3)	0.40082 (5)	0.0145 (3)	
C5	0.12296 (16)	1.0241 (2)	0.43419 (5)	0.0136 (3)	
C6	0.13589 (17)	1.1894 (3)	0.40729 (5)	0.0170 (3)	
H6	0.2272	1.2330	0.3992	0.020*	
C7	0.01603 (17)	1.2911 (3)	0.39217 (5)	0.0183 (4)	
H7	0.0241	1.4039	0.3738	0.022*	
C8	−0.11438 (17)	1.2240 (3)	0.40457 (5)	0.0177 (4)	
C9	−0.13193 (17)	1.0592 (3)	0.43024 (5)	0.0200 (4)	
H9	−0.2238	1.0148	0.4376	0.024*	
C10	−0.01149 (17)	0.9592 (3)	0.44518 (5)	0.0176 (3)	
H10	−0.0210	0.8450	0.4631	0.021*	
C11	0.45981 (17)	0.6634 (3)	0.36697 (5)	0.0157 (3)	
C12	0.56581 (17)	0.7042 (3)	0.33933 (5)	0.0158 (3)	
C13	0.65699 (19)	0.8859 (3)	0.33351 (6)	0.0245 (4)	
H13A	0.6911	0.8880	0.3049	0.037*	
H13B	0.7380	0.8799	0.3531	0.037*	
H13C	0.6021	1.0100	0.3387	0.037*	
C14	0.66475 (17)	0.4832 (3)	0.28258 (5)	0.0167 (3)	
C15	0.77454 (18)	0.3469 (3)	0.28951 (5)	0.0204 (4)	
H15	0.7917	0.2913	0.3165	0.025*	
C16	0.85941 (19)	0.2924 (3)	0.25663 (6)	0.0229 (4)	
H16	0.9354	0.1995	0.2613	0.027*	
C17	0.83530 (18)	0.3714 (3)	0.21690 (5)	0.0207 (4)	
C18	0.72555 (19)	0.5103 (3)	0.21112 (6)	0.0260 (4)	
H18	0.7092	0.5685	0.1844	0.031*	
C19	0.63922 (19)	0.5658 (3)	0.24361 (5)	0.0234 (4)	
H19	0.5635	0.6594	0.2391	0.028*	
C20	0.9250 (2)	0.3050 (3)	0.18119 (6)	0.0324 (5)	
H20A	1.0108	0.2375	0.1920	0.049*	
H20B	0.9514	0.4247	0.1648	0.049*	
H20C	0.8710	0.2096	0.1635	0.049*	
H1N	0.5662 (19)	1.407 (3)	0.4648 (6)	0.020 (5)*	
H2N	0.574 (2)	1.255 (3)	0.4297 (7)	0.027 (6)*	

### Atomic displacement parameters (Å^2^)

**Table:**

	*U*^11^	*U*^22^	*U*^33^	*U*^12^	*U*^13^	*U*^23^
S1	0.0194 (2)	0.0157 (2)	0.0149 (2)	−0.00300 (17)	0.00448 (15)	−0.00283 (16)
F1	0.0177 (5)	0.0359 (6)	0.0223 (5)	0.0063 (5)	−0.0003 (4)	0.0081 (5)
N1	0.0174 (7)	0.0180 (8)	0.0191 (8)	−0.0041 (6)	0.0050 (6)	−0.0068 (6)
N2	0.0125 (6)	0.0151 (7)	0.0137 (7)	−0.0008 (5)	0.0024 (5)	−0.0030 (6)
N3	0.0149 (6)	0.0154 (7)	0.0132 (7)	0.0016 (6)	0.0022 (5)	−0.0015 (6)
N4	0.0287 (8)	0.0162 (7)	0.0190 (7)	−0.0038 (6)	0.0068 (6)	−0.0044 (6)
N5	0.0291 (8)	0.0177 (7)	0.0208 (8)	−0.0071 (6)	0.0080 (6)	−0.0044 (6)
N6	0.0203 (7)	0.0133 (7)	0.0147 (7)	−0.0028 (6)	0.0027 (5)	−0.0007 (6)
C1	0.0145 (7)	0.0131 (8)	0.0141 (8)	0.0020 (6)	−0.0026 (6)	0.0011 (6)
C2	0.0159 (8)	0.0118 (7)	0.0142 (8)	−0.0031 (6)	0.0014 (6)	0.0004 (6)
C3	0.0180 (8)	0.0137 (8)	0.0170 (8)	−0.0008 (7)	0.0025 (6)	−0.0006 (7)
C4	0.0155 (7)	0.0143 (8)	0.0138 (8)	0.0019 (6)	0.0001 (6)	0.0013 (6)
C5	0.0153 (7)	0.0136 (8)	0.0121 (7)	−0.0015 (6)	0.0011 (6)	−0.0023 (6)
C6	0.0150 (8)	0.0186 (8)	0.0174 (8)	−0.0042 (7)	0.0019 (6)	−0.0003 (7)
C7	0.0204 (8)	0.0179 (8)	0.0166 (8)	−0.0010 (7)	0.0004 (6)	0.0041 (7)
C8	0.0148 (8)	0.0230 (9)	0.0152 (8)	0.0036 (7)	−0.0012 (6)	0.0003 (7)
C9	0.0142 (8)	0.0270 (10)	0.0189 (8)	−0.0034 (7)	0.0033 (6)	0.0027 (7)
C10	0.0182 (8)	0.0187 (8)	0.0161 (8)	−0.0029 (7)	0.0017 (6)	0.0021 (7)
C11	0.0180 (8)	0.0132 (8)	0.0157 (8)	−0.0006 (6)	0.0001 (6)	−0.0010 (6)
C12	0.0188 (8)	0.0137 (8)	0.0150 (8)	0.0003 (7)	0.0000 (6)	−0.0032 (7)
C13	0.0273 (9)	0.0177 (9)	0.0290 (10)	−0.0070 (8)	0.0093 (8)	−0.0067 (8)
C14	0.0204 (8)	0.0151 (8)	0.0148 (8)	−0.0022 (7)	0.0033 (6)	−0.0033 (7)
C15	0.0252 (9)	0.0213 (9)	0.0148 (8)	0.0012 (7)	0.0008 (7)	0.0016 (7)
C16	0.0236 (9)	0.0239 (9)	0.0214 (9)	0.0059 (8)	0.0021 (7)	0.0015 (8)
C17	0.0239 (9)	0.0207 (9)	0.0177 (8)	0.0020 (7)	0.0039 (7)	−0.0039 (7)
C18	0.0330 (10)	0.0292 (10)	0.0158 (9)	0.0075 (8)	0.0030 (7)	0.0024 (8)
C19	0.0272 (9)	0.0240 (9)	0.0192 (9)	0.0104 (8)	0.0020 (7)	0.0019 (8)
C20	0.0372 (11)	0.0379 (12)	0.0223 (10)	0.0130 (9)	0.0089 (8)	0.0000 (9)

### Geometric parameters (Å, º)

**Table:**

S1—C1	1.6873 (17)	C7—C8	1.374 (2)
F1—C8	1.3724 (19)	C7—H7	0.9500
N1—C1	1.329 (2)	C8—C9	1.371 (2)
N1—H1N	0.89 (2)	C9—C10	1.389 (2)
N1—H2N	0.86 (2)	C9—H9	0.9500
N2—C1	1.350 (2)	C10—H10	0.9500
N2—N3	1.3996 (18)	C11—C12	1.379 (2)
N2—C2	1.469 (2)	C12—C13	1.483 (2)
N3—C4	1.289 (2)	C13—H13A	0.9800
N4—N5	1.311 (2)	C13—H13B	0.9800
N4—C11	1.368 (2)	C13—H13C	0.9800
N5—N6	1.360 (2)	C14—C19	1.381 (2)
N6—C12	1.352 (2)	C14—C15	1.382 (2)
N6—C14	1.441 (2)	C15—C16	1.387 (2)
C2—C5	1.521 (2)	C15—H15	0.9500
C2—C3	1.539 (2)	C16—C17	1.392 (2)
C2—H2	1.0000	C16—H16	0.9500
C3—C4	1.506 (2)	C17—C18	1.388 (3)
C3—H3A	0.9900	C17—C20	1.505 (2)
C3—H3B	0.9900	C18—C19	1.387 (2)
C4—C11	1.449 (2)	C18—H18	0.9500
C5—C10	1.391 (2)	C19—H19	0.9500
C5—C6	1.392 (2)	C20—H20A	0.9800
C6—C7	1.391 (2)	C20—H20B	0.9800
C6—H6	0.9500	C20—H20C	0.9800
			
C1—N1—H1N	119.3 (12)	C8—C9—C10	118.08 (15)
C1—N1—H2N	119.6 (14)	C8—C9—H9	121.0
H1N—N1—H2N	119.4 (19)	C10—C9—H9	121.0
C1—N2—N3	120.50 (13)	C9—C10—C5	120.82 (16)
C1—N2—C2	126.55 (13)	C9—C10—H10	119.6
N3—N2—C2	112.60 (12)	C5—C10—H10	119.6
C4—N3—N2	106.88 (13)	N4—C11—C12	109.01 (14)
N5—N4—C11	108.96 (14)	N4—C11—C4	119.94 (15)
N4—N5—N6	106.73 (13)	C12—C11—C4	131.01 (16)
C12—N6—N5	111.69 (13)	N6—C12—C11	103.62 (14)
C12—N6—C14	129.31 (14)	N6—C12—C13	124.45 (15)
N5—N6—C14	119.00 (13)	C11—C12—C13	131.93 (16)
N1—C1—N2	116.57 (15)	C12—C13—H13A	109.5
N1—C1—S1	123.20 (13)	C12—C13—H13B	109.5
N2—C1—S1	120.23 (12)	H13A—C13—H13B	109.5
N2—C2—C5	111.32 (13)	C12—C13—H13C	109.5
N2—C2—C3	100.70 (12)	H13A—C13—H13C	109.5
C5—C2—C3	113.13 (13)	H13B—C13—H13C	109.5
N2—C2—H2	110.4	C19—C14—C15	120.95 (16)
C5—C2—H2	110.4	C19—C14—N6	119.95 (15)
C3—C2—H2	110.4	C15—C14—N6	119.05 (15)
C4—C3—C2	101.42 (13)	C14—C15—C16	119.14 (16)
C4—C3—H3A	111.5	C14—C15—H15	120.4
C2—C3—H3A	111.5	C16—C15—H15	120.4
C4—C3—H3B	111.5	C15—C16—C17	121.23 (17)
C2—C3—H3B	111.5	C15—C16—H16	119.4
H3A—C3—H3B	109.3	C17—C16—H16	119.4
N3—C4—C11	122.33 (15)	C18—C17—C16	118.19 (16)
N3—C4—C3	114.34 (14)	C18—C17—C20	121.20 (17)
C11—C4—C3	123.26 (15)	C16—C17—C20	120.61 (16)
C10—C5—C6	119.23 (15)	C19—C18—C17	121.34 (17)
C10—C5—C2	118.75 (15)	C19—C18—H18	119.3
C6—C5—C2	122.02 (14)	C17—C18—H18	119.3
C7—C6—C5	120.48 (15)	C14—C19—C18	119.14 (17)
C7—C6—H6	119.8	C14—C19—H19	120.4
C5—C6—H6	119.8	C18—C19—H19	120.4
C8—C7—C6	118.18 (16)	C17—C20—H20A	109.5
C8—C7—H7	120.9	C17—C20—H20B	109.5
C6—C7—H7	120.9	H20A—C20—H20B	109.5
C9—C8—F1	118.69 (14)	C17—C20—H20C	109.5
C9—C8—C7	123.19 (16)	H20A—C20—H20C	109.5
F1—C8—C7	118.12 (15)	H20B—C20—H20C	109.5
			
C1—N2—N3—C4	174.29 (14)	C6—C5—C10—C9	−1.1 (3)
C2—N2—N3—C4	−12.04 (17)	C2—C5—C10—C9	179.58 (15)
C11—N4—N5—N6	−0.27 (19)	N5—N4—C11—C12	0.2 (2)
N4—N5—N6—C12	0.23 (19)	N5—N4—C11—C4	−177.76 (15)
N4—N5—N6—C14	179.55 (14)	N3—C4—C11—N4	172.91 (15)
N3—N2—C1—N1	−0.5 (2)	C3—C4—C11—N4	−3.8 (2)
C2—N2—C1—N1	−173.19 (15)	N3—C4—C11—C12	−4.6 (3)
N3—N2—C1—S1	−179.93 (11)	C3—C4—C11—C12	178.72 (17)
C2—N2—C1—S1	7.3 (2)	N5—N6—C12—C11	−0.08 (18)
C1—N2—C2—C5	72.6 (2)	C14—N6—C12—C11	−179.32 (16)
N3—N2—C2—C5	−100.65 (15)	N5—N6—C12—C13	179.88 (16)
C1—N2—C2—C3	−167.24 (15)	C14—N6—C12—C13	0.6 (3)
N3—N2—C2—C3	19.54 (16)	N4—C11—C12—N6	−0.08 (18)
N2—C2—C3—C4	−18.17 (15)	C4—C11—C12—N6	177.61 (17)
C5—C2—C3—C4	100.72 (15)	N4—C11—C12—C13	179.96 (18)
N2—N3—C4—C11	−178.76 (14)	C4—C11—C12—C13	−2.4 (3)
N2—N3—C4—C3	−1.78 (18)	C12—N6—C14—C19	−76.7 (2)
C2—C3—C4—N3	13.53 (18)	N5—N6—C14—C19	104.1 (2)
C2—C3—C4—C11	−169.51 (15)	C12—N6—C14—C15	106.0 (2)
N2—C2—C5—C10	−165.88 (14)	N5—N6—C14—C15	−73.2 (2)
C3—C2—C5—C10	81.57 (18)	C19—C14—C15—C16	−0.3 (3)
N2—C2—C5—C6	14.8 (2)	N6—C14—C15—C16	176.99 (16)
C3—C2—C5—C6	−97.72 (18)	C14—C15—C16—C17	−0.4 (3)
C10—C5—C6—C7	1.2 (2)	C15—C16—C17—C18	1.4 (3)
C2—C5—C6—C7	−179.49 (15)	C15—C16—C17—C20	−177.77 (18)
C5—C6—C7—C8	0.1 (3)	C16—C17—C18—C19	−1.7 (3)
C6—C7—C8—C9	−1.6 (3)	C20—C17—C18—C19	177.47 (19)
C6—C7—C8—F1	178.40 (15)	C15—C14—C19—C18	0.0 (3)
F1—C8—C9—C10	−178.27 (15)	N6—C14—C19—C18	−177.25 (16)
C7—C8—C9—C10	1.7 (3)	C17—C18—C19—C14	1.0 (3)
C8—C9—C10—C5	−0.3 (3)		

### Hydrogen-bond geometry (Å, º)

**Table:**

*D*—H···*A*	*D*—H	H···*A*	*D*···*A*	*D*—H···*A*
N1—H1*N*···S1^i^	0.89 (2)	2.432 (19)	3.3159 (14)	172.7 (16)
N1—H2*N*···F1^ii^	0.86 (2)	2.29 (2)	2.9940 (18)	138.9 (17)
N1—H2*N*···N3	0.86 (2)	2.30 (2)	2.6554 (19)	104.9 (15)
C3—H3*A*···S1^iii^	0.99	2.87	3.8390 (19)	166
C9—H9···S1^iv^	0.95	2.83	3.5595 (18)	135
C15—H15···F1^v^	0.95	2.41	3.2502 (19)	148

Symmetry codes: (i) −*x*+1, −*y*+3, −*z*+1; (ii) *x*+1, *y*, *z*; (iii) *x*, *y*−1, *z*; (iv) −*x*, −*y*+2, −*z*+1; (v) *x*+1, *y*−1, *z*.
